# Differential traits between microvesicles and exosomes in enterovirus infection

**DOI:** 10.1002/mco2.384

**Published:** 2023-09-24

**Authors:** Yuxuan Fu, Sidong Xiong

**Affiliations:** ^1^ Jiangsu Key Laboratory of Infection and Immunity Institutes of Biology and Medical Sciences Soochow University Suzhou China

**Keywords:** biodistribution, clinical diagnostics, enterovirus, exosomes, microvesiclesss

## Abstract

Extracellular vesicles (EVs), including exosomes and microvesicles (MVs), are released by most cell types into the extracellular space and represent the pathophysiological condition of their source cells. Recent studies demonstrate that EVs derived from infected cells and tumors contribute to disease pathogenesis. However, very few studies have rigorously characterized exosomes and microvesicles in infectious diseases. In this study, we focused on subpopulations of EVs during the human enterovirus infection and explored the distinct traits and functions of EVs. We construct an effective immunomagnetic method to isolate exosomes and MVs from enterovirus‐infected cells excluding virion. The morphology and sizes of exosomes and MVs have no significant alteration after enterovirus infection. Meanwhile, our study observed that the enterovirus infection could induce exosome secretion but not MVs. In vivo study showed that there was differential biodistribution between exosomes and MVs. Using deep RNA sequencing, we found that the cargo information in MVs rather than in exosomes could accurately reflect pathological condition of original cells. Our study demonstrated that it should be considered to use MVs as clinical diagnostics during in enterovirus infection because their composition is reflective of pathological changes.

## Graphical abstract

Host cells infected by enterovirus not only release the virion but also extracellular vesicles (EVs), including exosomes and microvesicles (MVs). Their exhibited differential tissue specificity with enriched biodistribution in liver, intestine, and kidney for exosomes while in liver, lung, and spleen for MVs. The biodistribution of exosomes and MVs from host cells have no alteration when infected by enterovirus.

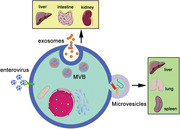

## 1 INTRODUCTION

Extracellular vesicles (EVs) are naturally released by healthy, morbid, and virus‐infected cells. They are specifically mediate intercellular communication through the transfer of nucleic acid, proteins, or lipids to recipient cells.^1^, ^2^ EVs charges carry specific cargo corresponding to the pathophysiological condition of their source cells, providing prognostic or diagnostic information to assess the disease progression.^3^ There is growing interest in their potential use as minimally invasive disease or predictive biomarkers. Therefore, understanding the interplay between viruses and EVs may contribute to an understanding of the molecular mechanisms that underlie these roles, the potential consequences for the infected host, and possible future diagnostic applications.

According to the different origin and size, EVs can be classified as either microvesicles (MVs) or exosomes. Exosomes are generally defined as 30–100 nm in size and formed as a result of multivesicular bodies (MVBs) containing intraluminal vesicles (ILVs) being trafficked from the cytosol to the plasma membrane. The MVBs then fuse with the cytomembrane, releasing ILVs, so‐called exosomes. On the contrary, MVs are sorted to be 200−1000 nm in diameter and released through direct outward budding and fission of the plasma membrane.^4^, ^5^ Both exosomes and MVs can loading and transfer specific cargo to recipient cells result in influencing cellular physiological condition.^6^, ^7^ Barberies et al.’s study investigated the exosomes as a diagnostic biomarker with high level of C‐reactive protein (CRP) in the SARS‐CoV‐19‐infected patients. The CRP was able to differentiate between positive patients and negative patients.^8^ Early‐stage detection of hepatocellular carcinoma was proposed based on a combination of exosomes with microRNA to overcome the inadequate diagnosis and poor sensitivity of Alpha‐fetoprotein (AFP) and ultrasound screening.^9^

In fact, many studies concentrated on the function of exosomes that released from cells infected with various viruses, including viral transmission, host immune response, and manipulation of the microenvironment. But little is known about the function of microvesicles in viral infection.^10^ Importantly, it is necessary to note that many researches still blur the difference with the term “exosomes/microvesicles.” For instance, vesicles isolated from biofluids using the same methods can be referred to as exosomes by some, and microvesicles by others.^5^, ^11^ Very few studies have rigorously distinguished between the two. Of note, because EVs are produced by virtually all cells, probably every viral preparation is in fact a mixture of virions and EVs. Given the ever‐growing roles and importance of EVs in viral infections, it is necessary to define biological concepts explaining differences in the characterization of EVs on infectious diseases.

Enteroviruses are a large genus of single positive‐stranded RNA viruses including poliovirus, Coxsackievirus, rhinovirus, and enterovirus 71 that can cause poliomyelitis, myocarditis, hand foot, and mouth disease and the common cold.^12^ However, little is known about the molecular cargo in different types of EVs from enterovirus‐infected cells, or the fate of EVs after they are generated and in the extracellular space. To address these issues, we report here differential traits and functions of exosomes and MVs upon enterovirus infection by in vivo and in vitro experiments. We found that the subpopulation of EVs could be efficiently isolated through the immunomagnetic method from enterovirus‐infected cells. Enterovirus infection‐induced increase of exosome secretion rather than MVs in human intestinal cells. In vivo studies showed that the distribution of exosomes and MVs had different tissue specificity. Importantly, our data demonstrated that the MVs‐contained contents could accurately reflect pathological conditions of original cells rather than exosomes during enterovirus infection. Our findings provide evidence that microvesicles are a more appropriate tool for clinical diagnostics and prognostics during enterovirus infection because their composition is reflective of pathological changes.

## 2 RESULTS

### 2.1 Characterization of exosomes and microvesicles from Coxsackievirus B3‐infected cells

**FIGURE 1:**
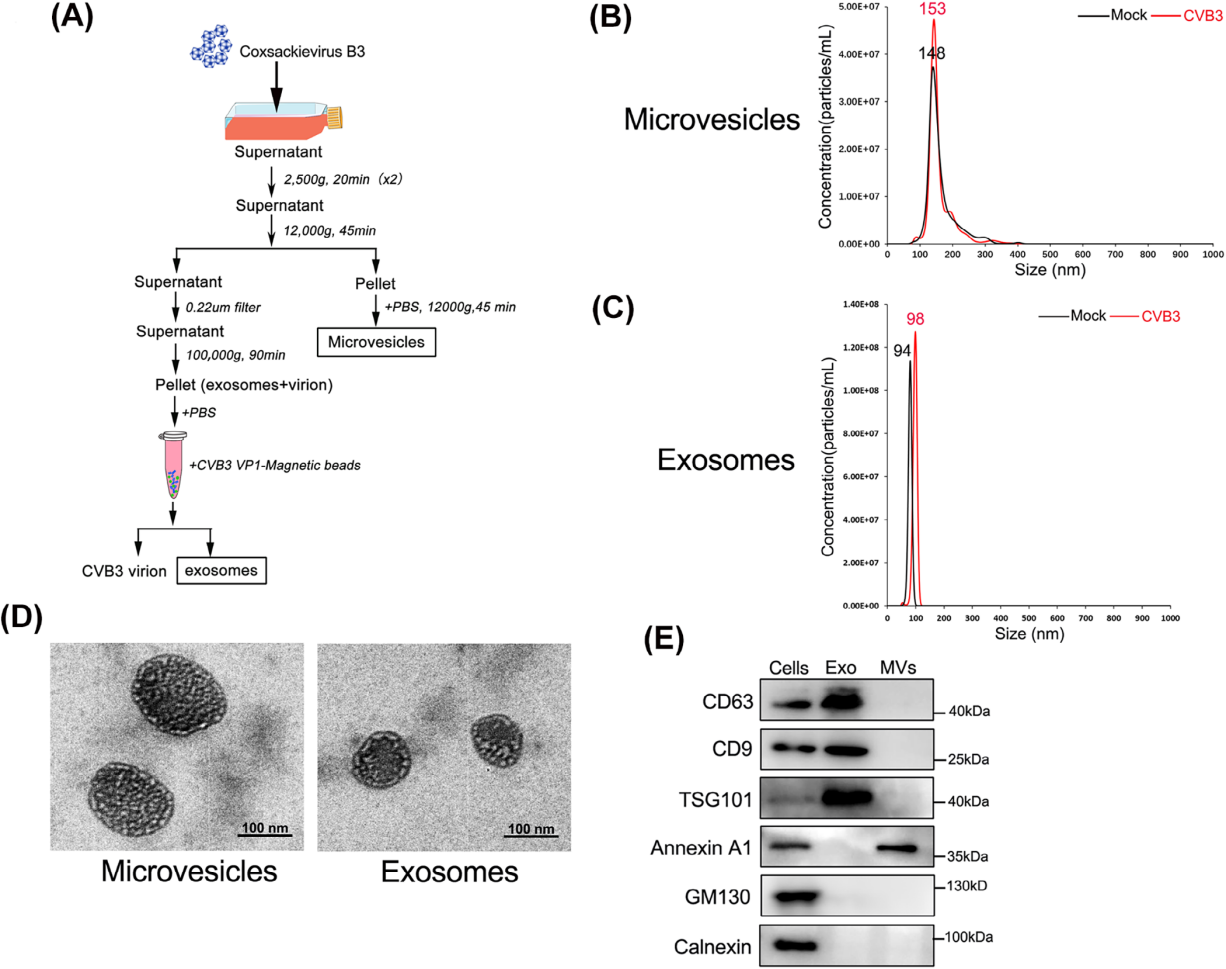
Characterization of exosomes and microvesicles from enterovirus‐infected cells. (A) Schematic presentation of isolation procedure to separate microvesicles (MVs), purified exosomes, and virion from CVB3‐infected HCT‐116 cells. (B and C) The size of microvesicles and purified exosomes from mock or CVB3‐infected cells was determined by the nanoparticle tracking analysis (NTA). (D) The electron micrographs of microvesicles and purified exosomes from CVB3‐infected cells were analyzed by Transmission electron microscopy. (E) Purified exosomes and MVs isolated from infected cells above described were determined by western blot using exosomal markers CD9, CD63, and TSG101, MVs markers Annexin A1, Golgi marker GM130 and ER marker calnexin. The lysate of CVB3‐infected cell as positive control.

We isolated EVs from the culture medium of Coxsackievirus B3 (CVB3)‐infected HCT‐116 cells (human intestinal epithelial cells) through differential ultracentrifugation (Figure 1A) as previously described with modifications. Because CVB3 virions and exosomes have a close density, we optimized the differential ultracentrifugation combined with immunomagnetic beads to isolate purified exosomes from culture medium of CVB3‐infected cells. The identity of the studied vesicles was confirmed by nanoparticle tracking analysis (NTA). The NTA showed the predominant peak size of MVs from CVB3‐infected cells was 163 nm, similar to mock MVs with a peak of 158 nm (Figure 1B). Meanwhile, the exosomes from CVB3‐infected cells showed a size of 98 nm which similar to mock exosomes with peak sizes of 94 nm (Figure 1C). These results suggested that CVB3 infection did not alter the size of exosomes or MVs. Of note, the predominant peak size in the NTA data does not represent the mean size of MVs or exosomes, and the diameter of MVs or exosomes exhibited diversity. Moreover, the electron micrographs of the MVs or exosomes from CVB3‐infected cells revealed rounded structures with the size of approximately 150 and 100 nm, respectively, which is consistent with typically accepted properties (Figure 1D). In addition, western blot was also used to analyze the purified exosomes and MVs of cell culture supernatants from CVB3‐infected HCT‐116 cells. Then we found the high enrichment of exosomal markers CD63, CD9, and TSG101 was present in purified exosome fraction, and Annexin A1 represents a good microvesicle marker (Figure 1E). All the vesicles were negative for GM130 (Golgi matrix marker), calnexin (endoplasmic reticulum marker), and virus‐structure protein (VP1), indicating the immunomagnetic method could efficiently isolate subpopulation of EVs from virion contamination.

### 2.2 Enterovirus infection induced exosome secretion rather than microvesicles

**FIGURE 2:**
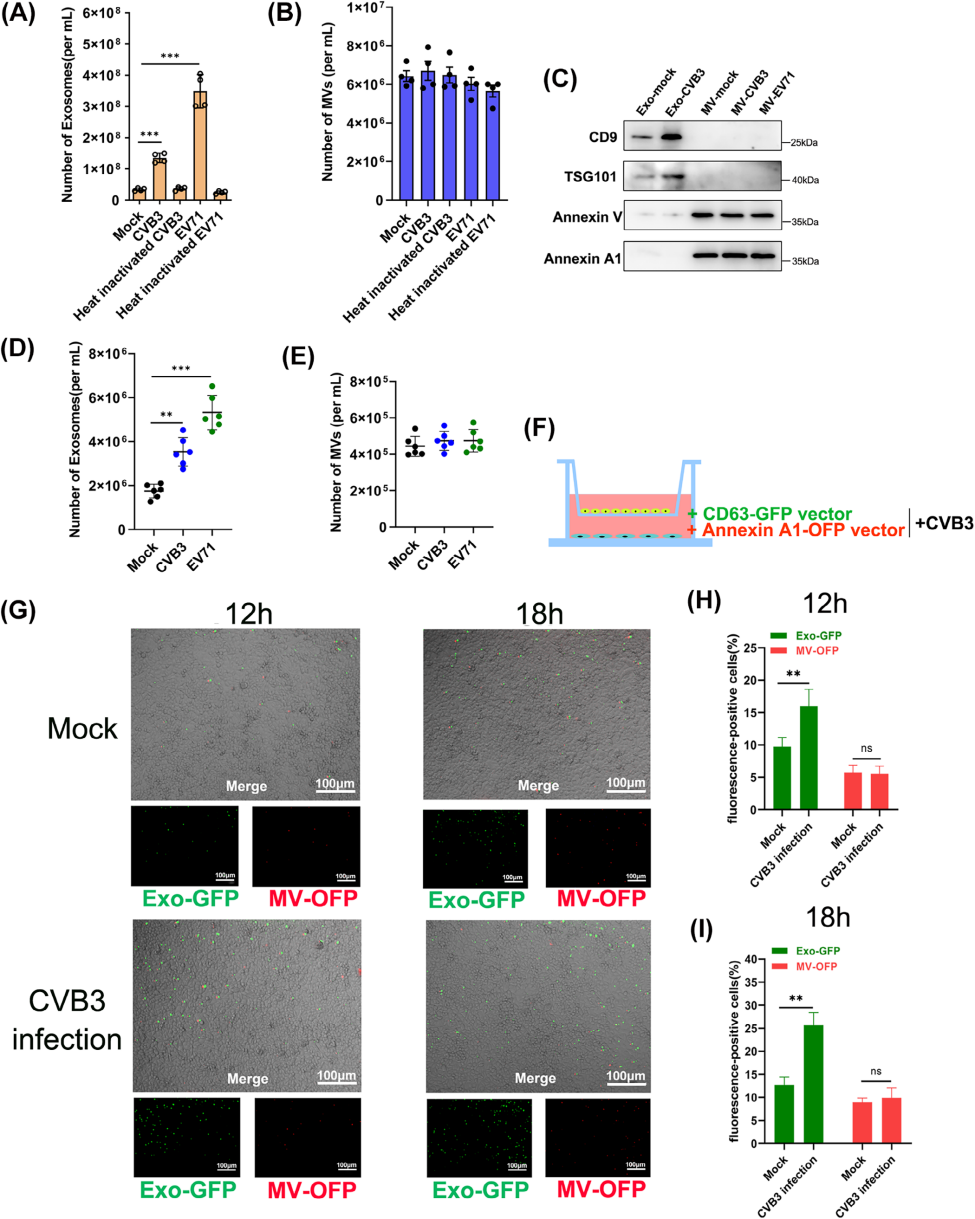
Enterovirus infection induced exosome secretion rather than microvesicles. (A–C) Quantification of exosomes and MVs purified from HCT‐116 cells with different infections was determined using nanoparticle tracking analysis (A and B) and Western blot (C). Data are shown as mean ± SD of four independent experiments. (***p < 0.001). (D and E) The purified exosomes and MVs were isolated from serum of CVB3‐infected, EV71‐infected, or uninfected individuals and quantified using NTA. (*n* = 6). Data are shown as mean ± SD (**p < 0.01, ***p < 0.001). (F) Schematic presentation of the transwell coculture with HeLa cells in the top well and treatment of HCT‐116 cells in the bottom well. The HCT‐116 cells were cotransfected with CD63‐GFP and Annexin A1‐OFP vectors, and then infected with CVB3 at a 0.05 TCID_50_ before coculture. A porous (1 μm) membrane allows transfer of exosomes and MVs but precludes direct cell‐cell contact. (G) Fluorescent images of HeLa in the top well were captured at 12 and 18 h after coculture with HCT‐116 cells. Bar = 100 μm. (H and I) Flow cytometric analysis of GFP and OFP ratio in HeLa cells after coculture with HCT‐116 cells at 12 and 18 h. Data are shown as mean ± SD of three independent experiments. (**p < 0.01, ns: nonsignificant).

Previous study demonstrated that EV71 infection upregulated exosome production.^13^ Similarly, we observed that CVB3‐infected HCT‐116 cells also had a significant increase of exosomes secretion compared to the mock cells or heat‐inactivated CVB3 treated cells as determined by NTA (Figure 2A). Unexpectedly, the MVs secretion showed no significant difference between virus‐infected cells and mock cells (Figure 2B). Western blot was also used to analysis the purified exosomes and MVs of cell culture supernatants from equal number of HT‐29 cells with or without infecting by enterovirus. Then we found the exosomal markers CD9 and TSG101 in exosomes fraction from CVB3‐infected cells (Exo‐CVB3) were significantly higher than that from mock cells (Exo‐Mock) (Figure 2C). The Annexins V and A1, the accepted MVs marker proteins, were showed no significant difference in MVs fraction, which, respectively, came from CVB3 (MV‐CVB3), EV71 (MV‐EV71), and mock cells, consistent with results of NTA. These results were also supported by mice data as we quantified the exosomes and MVs of enterovirus‐infected mice serum. Significantly, higher levels of exosomes but not MVs were found in the sera of infected mice than the uninfected, as shown in Figure 2D and E.

To further quantify the distinct EVs secretion, we performed the HCT‐116 cells cotransfected with CD63‐GFP and Annexin A1‐OFP fused expression vectors before infecting with CVB3, and then cocultured with HeLa cells in transwell chambers with 1.0 μm pore size (Figure 2F). The percentage of green‐fluorescence‐positive HeLa cells was significantly higher than the uninfected cells by immunofluorescence and flow cytometry at 12 and 18 h, while the red‐fluorescence‐positive cells shown no significant difference at indicated time points compared to uninfected cells (Figure 2G–I), suggesting that enterovirus infection could induce exosome secretion rather than MVs.

### 2.3 Exosomes and microvesicles exhibited differential biodistribution in vivo

**FIGURE 3:**
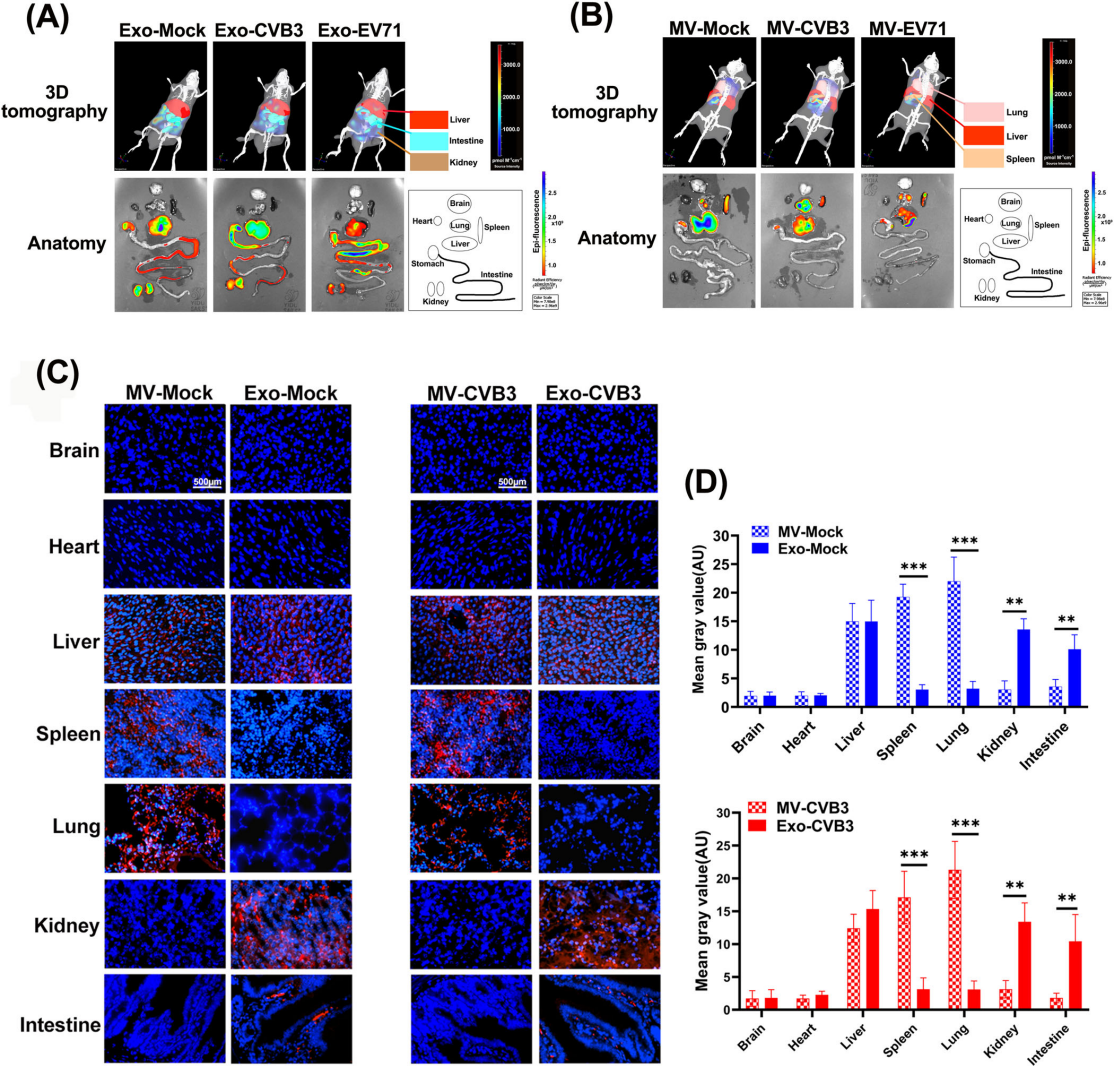
Exosomes and microvesicles exhibited differential biodistribution in vivo. (A and B) The representative IVIS images of different organs were acquired through intravenous injection of exosomes (A, Exo‐EV71, Exo‐CVB3) or MVs (B, MV‐EV71, MV‐CVB3) from enterovirus‐infected cells. Exosomes (Exo‐Mock) or MVs (MV‐Mock) from mock cells as the control. Each mouse was injected with 150 μg DiD‐labeled exosomes or MVs for 24 h, and then animals were euthanized sacrificed for tissue collection. Radiant efficiency was measured using Living Image 3.1 software. (C and D) Fixation of the various organ from mice treated with DiD‐labeled exosomes or MVs for 24 h. After tissue sectioning, representative images were acquired by confocal microscope. The determination of mean gray value (AU) was used Image J software from six different fields.

Extracellular vesicles released from virus‐infected cells contain various viral and host components that are able to affect recipient host cell process.^10^ To further characterize the tissue distribution of exosomes and microvesicles that from virus‐infected cells in vivo, fluorescent dye DiD‐labeled exosomes (Exo‐CVB3, Exo‐EV71) or MVs (MV‐CVB3, MV‐EV71) isolated from enterovirus‐infected HCT‐116 cells were injected into tail veins of mice. Exosomes (Exo‐Mock) or MVs (MV‐Mock) from mock HCT‐116 cells are the control. In vivo fluorescence imaging exhibited that strong signals were detected in liver, intestine, and kidney when injected with Exo‐CVB3 or Exo‐EV71 at 24 h postinjection, similar to the injection of Exo‐Mock (Figure 3A). However, the main fluorescence signals were appeared in liver, lung, and spleen after injecting with MV‐CVB3, MV‐EV71, and MV‐Mock (Figure 3B). These observations suggested that the biodistribution of exosomes and MVs had the tissue specificity in vivo, and enterovirus infection did not alter this tissue localization. These observations were further confirmed by tissue sections. DiD‐labeled Exo‐Mock and Exo‐CVB3 were enriched in the intestine and kidney than animals injected with MV‐Mock or MV‐CVB3, in which higher fluorescence signals were identified in the lung and spleen (Figure 3C and D).

### 2.4 Enterovirus infection did not alter the expression levels of surface proteins for exosomes and microvesicles

**FIGURE 4:**
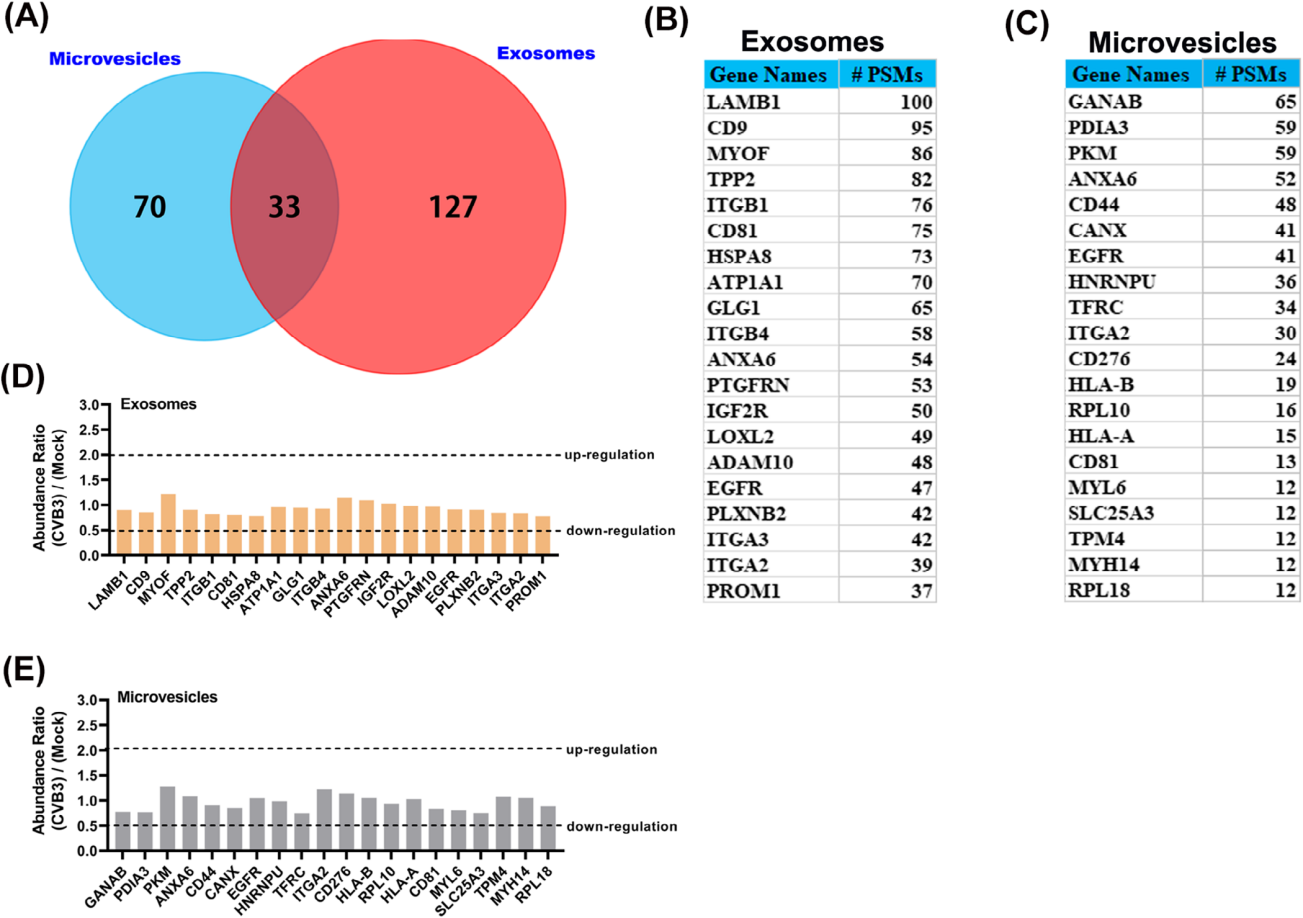
Enterovirus infection did not alter the expression levels of surface proteins for exosomes and microvesicles. (A) Venn diagram of membrane protein in exosomes and MVs though tandem mass spectrometry (LC‐MS/MS). (B and C) The top enrichment of 20 membrane proteins in exosomes and MVs was listed. #PSMs: number of spectrum which protein matched. (D and E) The abundance ratio for top enrichment of 20 membrane proteins in exosomes (D) and MVs (E) from mock and CVB3‐infected cells as determined by LC‐MS/MS.

To explore the mechanism of differential biodistribution on exosomes and microvesicles, we sought to characterize the composition of membrane protein in exosomes and MVs though tandem mass spectrometry (LC‐MS/MS). As shown in Figure 4A, 160 and 103 membrane proteins were detected in exosomes and MVs, respectively. Moreover, only 20% of proteins in exosomes also existed in MVs, indicating that the distinct composition of membrane proteins may contribute to the biodistribution of exosomes and MVs. In addition, we respectively listed the top enrichment of 20 membrane proteins in exosomes (Figure 4B) and MVs (Figure 4C). The LC‐MS/MS data showed that there was no significant alteration of these top proteins in exosomes from CVB3‐infected cells compared to mock exosomes (Figure 4D); similar result was also found in MVs (Figure 4E). These results demonstrated that enterovirus infection did not alter the expression levels of surface proteins for exosomes and microvesicles.

### 2.5 Microvesicles containing protein‐coding RNA profiles effectively reflected the pathological response of parental cells

**FIGURE 5:**
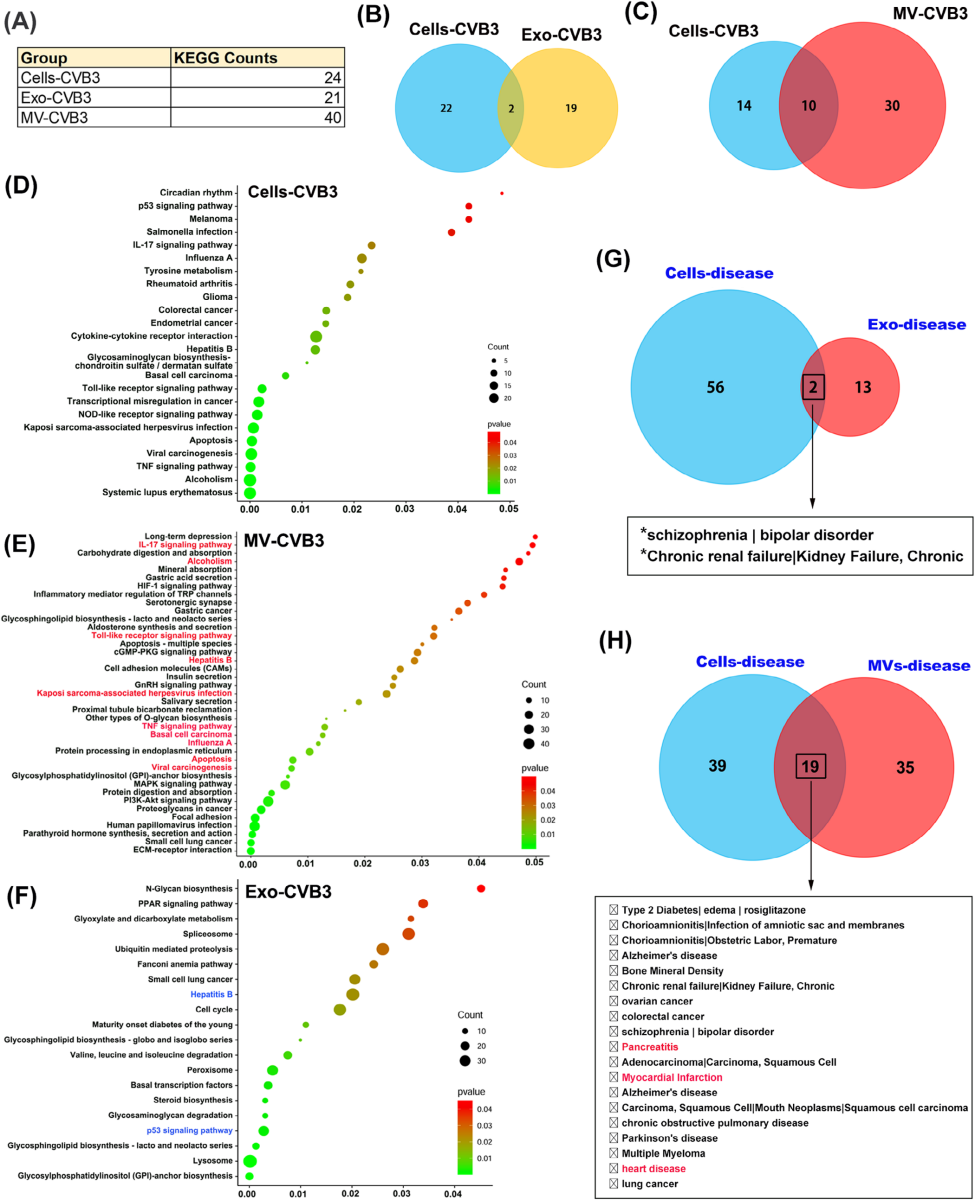
Microvesicle containing protein‐coding RNA profile effectively reflected the pathological response of parental cells. (A–C) KEGG pathway analysis of significantly changed protein‐coding RNAs in CVB3‐infected cells (Cells‐CVB3) as well as cell‐derived exosomes (Exo‐CVB3) or MVs (MV‐CVB3) using DAVID Bioinformatics Resources. HCT‐116 cells were infected with CVB3 for 24 h, then the cells, secreted exosomes, and MVs were collected for RNA‐seq sequencing. Three replicate samples in each group were performed. The KEGG pathway of Cells‐CVB3 vs. Exo‐CVB3 and Cells‐CVB3 vs. MV‐CVB3 were also shown by the Venn diagram (B and C). (D–F) KEGG pathway analysis of significantly changed protein‐coding RNAs in CVB3‐infected cells (Cells‐CVB3), cell‐derived exosomes (Exo‐CVB3), and MVs (MV‐CVB3) using DAVID Bioinformatics Resources. The red font represents the overlapping pathway of Cells‐CVB3 and Exo‐CVB3, while the blue font represents the overlapping pathway of Cells‐CVB3 and MV‐CVB3. (G and H) The significantly changed protein‐coding RNAs of infected cells, derived exosomes, and MVs were analyzed by the GAD_DISEASE tool in the DAVID Bioinformatics Resource. Venn diagrams of known disease of infected cells, exosomes, and MVs were presented. The overlap disease items were listed.

Previous study has now shown that the sorting of RNAs into EVs was selected, and EV RNA signature could reflect the biological activities of the parental cells.^14^ To investigate EV RNA contents that were indeed representative of parental cells, protein‐coding RNA (mRNA) profiles in the exosomes and MVs fractions from CVB3‐infected HCT‐116 cells were analyzed. We found that 3741 and 1859 mRNA transcripts were identified as significantly changed in exosomes and MVs, respectively. Meanwhile, 889 mRNA transcripts were also significantly changed in parental cells after CVB3 infection. The KEGG pathway analysis of changed mRNA transcripts of exosomes, MVs, and CVB3‐infected cells was mapped using the DAVID Bioinformatics Resource. We found that there were 24 pathways significantly enriched in CVB3‐infected cells (Cells‐CVB3). About 21 significant pathways were enriched in exosomes (Exo‐CVB3) and 40 enriched pathways in MVs (MV‐CVB3) (Figure 5A). The Venn diagram showed that 2 enriched pathways in exosomes and 10 pathways in MVs were overlapped with the identified pathways of CVB3‐infected parental cells (Figure 5B and C). Importantly, the significant enriched KEGG pathways in both Cells‐CVB3 and MV‐CVB3 were associated with virus infection (Influenza A, Hepatitis B, KSHV infection), inflammatory response (IL‐17 signaling pathway, TLR signaling pathway, TNF signal pathway), and apoptosis signal pathways in MV‐CVB3, consistent with CVB3‐induced pathology in the host (Figure 5D and E). Only two KEGG pathways (Hepatitis B and p53 signaling pathway) were enriched in Cells‐CVB3 and Exo‐CVB3 (Figure 5F). These results suggested that MVs may effectively represent the pathological state of enterovirus infection in source cells rather than exosomes.

To identify any relationships between these mRNAs and disease in exosomes or MVs, we analyzed associations between changed mRNA transcripts and known disease‐related genes using the GAD_DISEASE tool in the DAVID Bioinformatics Resource. As shown in Figure 5G and H, multiple diseases identified in MVs fraction, including pancreatitis, myocardial infarction, and heart disease, which were associated with CVB3‐induced clinical pathology. However, we found that enriched disease in exosomes was poorly related to the disease of CVB3 infection. Taken together, these data suggested that MV‐containing mRNA contents could exhibit the original host cells pathological conditions.

### 2.6 The noncoding RNA profile in MVs related to the condition of parental cells

**FIGURE 6:**
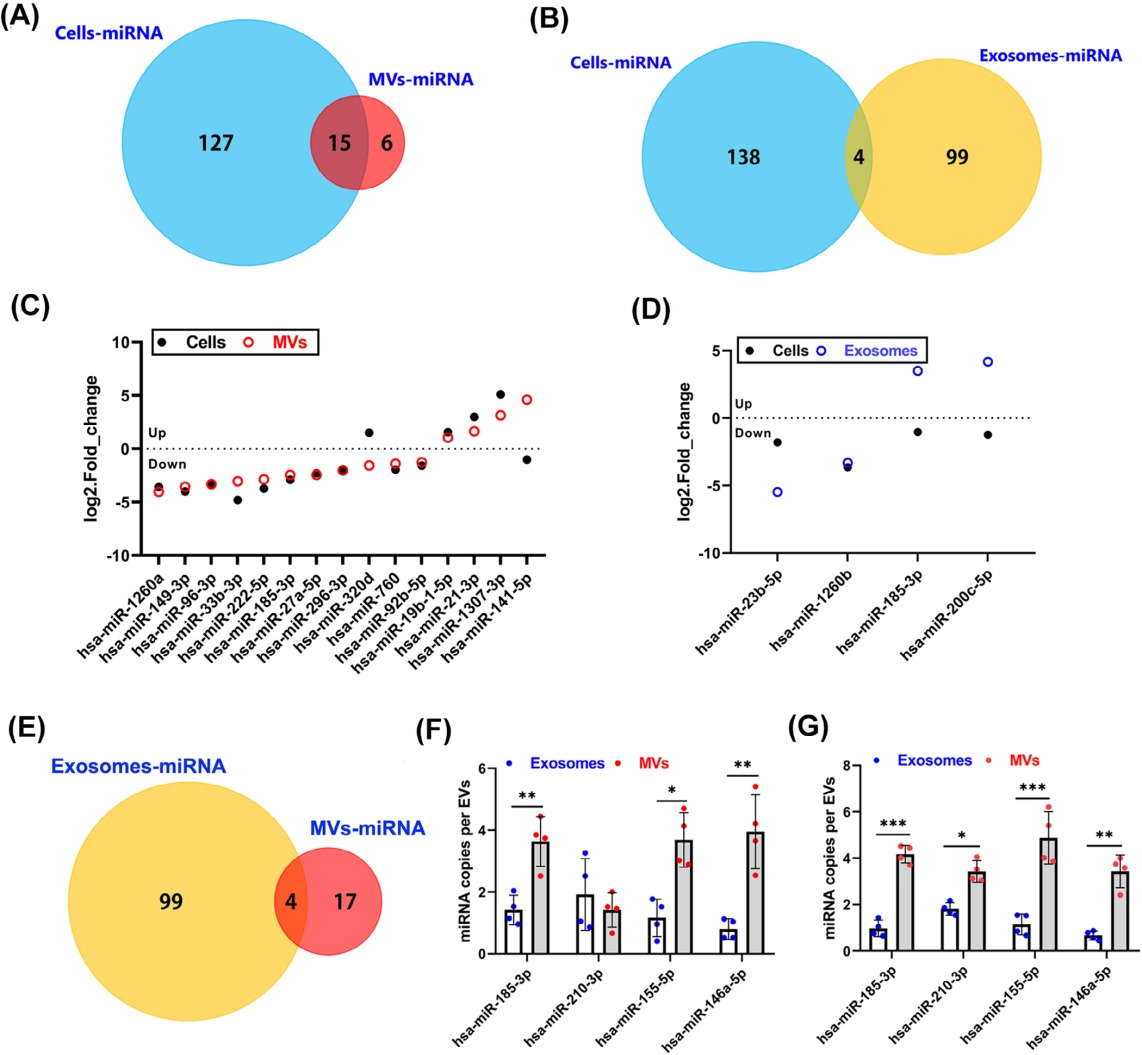
The noncoding RNA profile in MVs related to the condition of parental cells. (A and B) Venn diagram of significantly changed miRNAs from CVB3‐infected cells (Cells‐miRNA), cell‐derived MVs (MVs‐miRNA), and exosomes (Exosomes‐miRNA). (C and D) The alteration tendency of changed miRNAs in infected cells, derived exosomes, and MVs according to small RNA‐sequencing data. (E) Venn diagram of significantly changed miRNAs from CVB3‐infected cells derived MVs and exosomes. (F and G) Real‐time PCR analysis of miRNA copy numbers in exosomes and MVs that derived from HCT‐116 cells or Caco‐2 cells. Data are shown as mean ± SD of four independent experiments (*p < 0.05, **p < 0.01, ***p < 0.001).

A previous study demonstrated that the microRNAs (miRNA) were the most abundant RNA in EVs^15^; therefore, we performed a comprehensive microRNA profiling in exosomes and MVs isolated from CVB3‐infected HCT‐116 cells. Based on the significance of variation as indicated by ANOVA analysis, a total 142, 103, and 21 miRNAs were identified to be changed in virus‐infected cells, exosomes, and MVs, respectively, as shown in the Venn diagram (Figure 6A and B). About 15 (71.4%) of changed miRNAs in MVs overlapped with the identified miRNAs in original cells, while only 4 (3.8%) changed miRNAs in exosomes were also presented in infected cells (Figure 6C). Moreover, the most overlapped miRNAs (13 out of 15 miRNAs) in between cells and MVs had the same variation trend, but only two miRNAs in exosomes showed the same alteration tendency that consisted with virus‐infected cells (Figure 6D). These results indicated that MVs‐contained miRNAs may accurately reflect intracellular miRNA contents changed during virus infection than exosomes. In addition, to investigate the levels of miRNAs in different types of EVs, we chose four miRNAs that present both in MVs and exosomes according to the RNA‐seq data (Figure 6E). As shown in Figure 6F, three miRNAs (miR‐185‐3p, miR‐155‐5p, miR‐146‐5p) in HCT‐116 cell‐derived MVs showed higher copy number than those in exosomes. Meanwhile, MVs derived from Caco‐2 cells also showed significantly higher copy numbers of these four miRNAs than exosomes, suggesting that the MVs contained more enrichment of miRNA contents than exosomes (Figure 6G). Taken together, these results revealed that the MVs exhibited pathological conditions of original cells and may use for clinical diagnostics and prognostics during enterovirus infection.

## 3 DISCUSSION

Extracellular vesicles (EVs) secreted from virus‐infected cells not only contain cellular proteins and RNAs but also viral proteins and genetic material.^10^, ^16^ Although it is widely accepted that EVs are highly heterogeneous in size, structure, and biogenesis, currently they still lack sufficient practical nomenclature. Most commonly, EVs are generated inside cells and, on release into the extracellular milieu, are called “exosomes,” whereas others pinch off from the plasma membrane and are generally referred to as “microvesicles.”^4^, ^17^ Much of our current understanding of the functions of exosomes released from infected cells harbor and deliver many regulatory factors including viral RNA and proteins, and another host functional biological elements to neighboring cells, helping to establish productive infections and modulating cellular responses. However, very little is known about the function of microvesicle‐mediated cargo transfer, especially the interrelation between exosomes and microvesicles during virus infection.^18^

In this study, we concentrated on the EVs participation during the human enterovirus infection and reinforced the distinct traits and functions of exosomes and MVs. We first performed a differential ultracentrifugation procedure as previously described and well studied to obtain MVs and exosomes from enterovirus‐infected HCT‐116 cells to compare these EVs.^19^, ^20^, ^21^ However, due to enteroviral virions and exosomes having a close density, ultracentrifugation (100,000 × *g*) caused aggregation of exosomes and virions, namely “crude exosomes.” We demonstrated the separation of purified exosomes from crude pellets by using virus‐specific antibodies combined with immune affinity beads to exclude virion contamination. NTA and TEM showed that MVs were larger and more heterogeneous in size than exosomes, consistent with many previous studies. Although several types of research revealed the size alone cannot be used to distinguish these EVs from one another,^22^ the isolated method by differential ultracentrifugation was still an efficient way for separation of exosomes and MVs at present because the density of the MV fraction is greater than that of the exosome fraction.

Previous study observed that EV71 infection could induce exosomes secretion.^13^ In this study, we showed that both CVB3 and EV71 infection upregulated exosome production rather than MVs in vivo and in vitro. Many studies reported that EV71 or CVB3 infection triggered the autophagy process, resulting in autophagosomes formation in host cells.^23^, ^24^, ^25^, ^26^ Current evidence demonstrated that the intracellular autophagosomes accumulation contributed to exosome release.^27^, ^28^ Therefore, we speculate the increased exosomes secretion may be due to enterovirus‐induced autophagy response. Further experiments need to be test.

Extracellular vesicles or exosomes released from virus‐infected cells always contain a variety of viral and host cellular factors that are able to modify recipient host cell responses.^10^ In this context, it would be interesting to determine the difference of exosome‐ or MV‐mediated transfer in vivo. Our study found that DiD‐labeled exosomes enriched in liver, intestine, and kidney, distinct from the animals injected with MV, in which high fluorescence signal was identified in liver, lung, and spleen. Our data supported the hypothesis that exosomes or MVs have a distinct organ‐specific targeting resulted in exhibiting differential tissue accumulation. This organ specificity may depend on surface proteins or membrane lipids of EVs when EVs were taken up by recipient cells though binding with plasma membrane. Indeed, MVs composition has been found to be enriched in cell‐surface proteins of the parental cell in terms of receptors and adhesion molecules,^29^ while exosomes contain a subset of proteins related to their endosomal origin and involved in MVBs formation.^30^, ^31^ Moreover, previous study showed that exosomes and MVs differed in their types of protein and lipid composition by high‐resolution lipidomic and proteomic analyses in three different cell types.^32^ In addition, we also found that enterovirus infection did not alter tissue localization of exosomes and MVs, indicating that the composition of surface proteins or membrane lipids on exosomes or MVs have no change during enterovirus infection. Indeed, the LC‐MS/MS data confirmed that the enrichment of 20 surface proteins for exosomes and microvesicles have no alteration after enterovirus infection. Still to now, very little is known about the function of MVs‐mediated cargo transfer. A study from Kanada et al. demonstrated that nucleic acids were differentially loaded into the exosomes and MVs and that only MVs could transfer functional plasmid DNA (pDNA) that leads to expression of reporter biomolecules.^22^ Nevertheless, the role of MVs in various pathological processes remained largely unexplored, in particular their function in vivo.

Nucleic acid signatures are the other important component of EVs, including both coding and noncoding RNAs, which reflected the physiological or pathological state of the parent cell.^2^ Moreover, many reports have shown that exosomal RNA profiles were altered under different physiological conditions.^33^, ^34^, ^35^, ^36^ In the current study, we investigated distinct RNA profiles in subpopulations of EVs from enterovirus‐infected cells using RNA‐sequencing analysis. Although Exo‐CVB3 fraction shown more abundant mRNA transcripts than MV‐CVB3 fraction, the KEGG analysis of mRNA being present in virus‐associated MVs provides biological information, associated with viral pathogenesis. Moreover, using the GAD_DISEASE tool in the DAVID Bioinformatics Resource demonstrated these significantly changed mRNA transcripts in MVs associated with CVB3‐induced clinical pathogenesis, such as pancreatitis, myocardial infraction, and heart disease, but poorly correlated associations in Exo‐CVB3. Previous study showed that there was a diverse collection of the exosomal RNA species among which microRNAs (miRNAs) were the most abundant with over 42% of all raw reads in human plasma samples.^15^ Our sequence analysis shown that exosomal miRNAs (103 miRNAs) were more abundant than in MVs (21 miRNAs) but only two changed miRNAs in exosomes overlapped with the identified miRNAs in CVB3‐infected cells. Moreover, most MV‐contained miRNAs showed a coincident alteration with that in virus‐infected cells, but poor consistency in exosomes. These findings suggested MVs contained miRNAs information, which provided valuable information with respect to viral pathogenesis than exosomes. Thus, MV miRNAs has shown potential to be used as noninvasive biomarkers to indicate disease states. Increasing evidence shown that profiled EV contained miRNAs in different samples, and some miRNAs can be used to aid in clinical diagnosis.^37^, ^38^, ^39^ Moreover, since quantitative PCR of the relatively stable miRNA population is a highly sensitive and specific method, diagnosis on the basis of MV miRNA in the serum is a promising. Nevertheless, our study lacked clinical samples and animal experiment to further confirm the potential application of microvesicles during enterovirus‐induced pathogenesis. Of note, the EVs that separated from clinical serum or other body fluids were complicated due to the source organs or tissues. Therefore, identification of specific EVs markers for different organ/tissue certainly can contribute to clinical application value of EVs

In conclusion, our findings altogether reinforce the characteristic and functions of exosomes and MVs during the human enterovirus infection. We found the distinct trait of exosomes and microvesicles not only in organ biodistribution but also in their contents. Importantly, the microvesicles, a role that was often overlooked, have the content information, which can accurately represent pathological conditions of enterovirus‐infected cells instead of exosomes. Our study demonstrated that it should be considered to use microvesicles as clinical diagnostics, at least in enterovirus infection.

## 4 MATERIALS AND METHODS

### 4.1 Cell lines and Viral strain

The cell lines HCT‐116, Caco‐2, 293T, and Vero E6 used were purchased from the Cell Bank of the Chinese Academy of Sciences. All cells were cultured in DMEM (Hyclone) supplemented with 10% (vol/vol) FBS. Coxsackievirus B3 Woodruff strain (GenBank: U57056.1) was propagated on Vero E6 cells. Virus titers were calculated as the 50% tissue culture infectious dose (TCID50) using the Reed‐Muench formula method.

### 4.2 Isolation and procedures for microvesicles, exosomes, and viral particles

The HCT‐116 cells were infected with CVB3 for 1 h at 0.05 TCID_50_. After washing with phosphate buffered saline (PBS), the cells changed into the DMEM supplemented with 10% EV‐depleted FBS (System Biosciences, #EXO‐FBS‐50A‐1) for the production of microvesicles, exosomes, or CVB3 virion. After 24 h, the culture medium was centrifuged at 2500 × *g* for 20 min to remove death cells and cell debris, then the medium was centrifuged at 16,000 × *g* for 45 min at 4°C to collect microvesicle pellet. Culture medium from the centrifugation step was passed through a filter (0.22 μm pore, Millipore) and then ultracentrifuged at 100,000 × *g* for 90 min (Beckman Coulter Optima XE‐100). Pellets were resuspended in PBS and incubated with CVB3 VP1‐specific antibody (Wako, # M706401‐2) for 4 h at room temperature. Then the PBS mixture was added the Protein A/G magnetic beads (MERCK‐millipore, #LSKMAGA02) for an additional 1 h at room temperature to isolate CVB3 particles. The tube placed on the magnetic stand to capture the beads resulted in collecting virion, transferred the supernatant to new centrifuge tube containing purified exosomes without virion.

### 4.3 Transmission electron microscope (TEM) and nanoparticle tracking analysis (NTA)

The size and concentration detection of exosomes and MVs was done using the NS300 machine (Malvern Instruments, Malvern, UK). The morphology of exosomes and MVs was carried out by TEM (Hitachi HT7700 TEM, Tokyo, Japan). All the experimental operations were described in our previous study.^40^

### 4.4 LC‐MS/MS analysis

LC‐MS/MS analysis for exosomes and MVs was carried out as previously described.^41^

### 4.5 Western blot

Cells or extracellular vesicles fraction were lysed in RIPA buffer (Santa Cruz, USA) and cleared lysates were collected by centrifugation at 12,000 × *g* for 10 min at 4°C. Lysates were boiled in 5 × gel loading buffer and separated by SDS‐PAGE gels. Proteins were transferred onto PVDF membranes (MERCK‐millipore) and detected with appropriate primary antibodies CD9 (Sigma, # SAB4503606), TSG101 (#49270, Signalway Antibody), GAPDH (Proteintech, #60004‐1), Annexin A5 (Abcam, #ab171870), Annexin A1 (Cell Signaling Technology, #32934), GM130 (Cell Signaling Technology, #12480), and Calnexin (Cell Signaling Technology, #2679) at 4˚C overnight. The HRP‐conjugated goat antimouse or rabbit IgG (Southern Biotech) was used and exposed using the chemiluminescence (ECL) (Millipore, #WBLUC0100). The band intensities were quantified by ImageJ software (NIH).

### 4.6 In vivo imaging

For EVs labeling, the DiD (Life Technologies, #V22887) was added into the PBS suspension of MV or exosomes with a final concentration for 1 μM. The 6‐week‐old male BALB/c mice were injected with DiD‐labeled MV or exosomes through the tail vein (150 μg MVs or exosomes per mouse). After 24 h, the mice were sacrificed, and the fluorescence of the whole body was acquired by IVIS spectrum (Caliper Life Sciences). The organs were prepared, and the red fluorescence was imaged in brain, heart, lung, liver, spleen, stomach, kidney, and intestine. The average radiant efficiency was calculated by using Living Image 3.1 software (Caliper Life Sciences). Organ sections were fixed in paraffin section and photographed using Nikon confocal microscope (Tokyo, Japan).

### 4.7 Small RNA sequencing and microRNA qPCR

Cells, microvesicles, and purified exososmes isolation described above was resuspended in RNAiso reagent (TaKaRa, #9108) for total RNA isolation following the manufacturer's instructions. The resulting RIN (RNA integrity number) scores and concentrations were considered when qualifying samples to proceed. The small RNA library construction and deep sequencing were described in previous study.^13^

For copy number analysis of microRNA in microvesicles or purified exosomes, RNA was reverse transcribed using a reverse‐transcription kit (TaKaRa, #RR047A) according to the manufacturer's instructions. cDNA was mixed with SYBR Green (Applied Biosystems) and specific primers of miRNA (RiBoBio, Guangzhou, China). The synthetic miRNA fragments input ranged from 1 fM to 100 pM to generate standard curves and reactions were run on ABI QuantStudio 6 Detection System.

### 4.8 Cell transfection and coculture

HCT‐116 cells were grown to 6‐well plate the day before transfection. The CD63‐GFP‐ and Annexin A1‐OFP‐fused expression vectors (purchased from Sino Biological Inc. China) transfection used Lipofectamine 3000 (Life Technologies, USA) according to the manufacturer's protocol. After 24 h, the HCT‐116 were infected with CVB3 for 1 h at 0.05 TCID_50_, then the cells were washed with PBS and coculture with HeLa cells using the transwell 1 μm pore membrane insert system (Corning) for 12 or 18 h. Fluorescence microscopy and images were analyzed using the Nikon confocal microscope (Tokyo, Japan). The percentage of fluorescence‐positive cells was determined by BD FACS Aria II.

### 4.9 Statistical analysis

Data were shown as means ± SD. All statistical analyses were performed using the GraphPad Prism 8.0. The unpaired Student's *t*‐test was used to compare differences between two groups, whereas comparison of multiple groups was performed using ANOVA with post hoc tests to compare differences between individual groups. Groups were considered significantly different when *p* < 0.05 (*p < 0.05; **p < 0.01; ***p < 0.001).

## AUTHOR CONTRIBUTIONS

YXF contributed to conceptualization, data curation, formal analysis, investigation, visualization, methodology, and writing‐original draft. YXF and SDX contributed to resources, supervision, funding acquisition, project administration, and writing‐review and editing. All authors have read and approved the final manuscript.

## CONFLICT OF INTEREST STATEMENT

The authors have no conflicts of interest to declare.

## ETHICS STATEMENT

All animal experimental protocols were approved by the Soochow University Animal Care Committee and research protocols were conducted in accordance with the animal behavioral guidelines, using approved protocols from the institutional animal care committee (#202210A0142).

## Data Availability

The data included in this study are available from the corresponding author on reasonable request.
